# Therapy-related second malignant neoplasms on top of neuroblastoma: frequency, types and risk factors

**DOI:** 10.1007/s12672-025-02661-6

**Published:** 2025-05-21

**Authors:** Mohamed Fawzy, Nihal Abdelfattah, Menna Alaa, Inas Mohsen, Sonya Soliman, Sherine Salem, Wael Zekri, Omar Arafah

**Affiliations:** 1 Department of Pediatric Oncology, Children’s Cancer Hospital Egypt-57357, Cairo, Egypt; 2 Department of Pediatric Oncology, National Cancer Institute, Cairo University, Cairo, Egypt; 3 Department of Clinical Research, Children’s Cancer Hospital Egypt-57357, 1 Seket Al-Emam Street, El-Madbah El-Kadeem Yard, El Saida Zenab, Cairo, 11617 Egypt; 4 Department of Clinical Pathology, Children’s Cancer Hospital Egypt-57357, Cairo, Egypt; 5 Department of Clinical Pathology, National Cancer Institute, Cairo University, Cairo, Egypt

**Keywords:** Treatment related, Secondary malignant neoplasms, Neuroblastoma

## Abstract

**Objectives:**

The aim of the study was to evaluate the long-term effect of multi-modal, risk-based treatment protocols on the development of treatment-related secondary malignant neoplasm (SMN) in patients during or after treatment of Neuroblastoma.

**Material and methods:**

This retrospective study included all patients with neuroblastoma treated at Children’s Cancer Hospital-Egypt from July 2007 to December 2022.

**Results:**

24 out of 2290 patients (1%) received risk-tailored multimodal treatment protocols suffered from either hematological (21/24) or solid (3/24) treatment-related SMN during or after treatment of their primary neuroblastoma disease. Age at neuroblastoma diagnosis ranged from 6 mo to 9.5 y (median age: 2 y) with male to female ratio of 1.2:1. Time to development of hematological treatment-related SMN was 14 mo to 8.3 y (mean: 3.7 y) versus 5.5–9.2 y (mean: 7.6 y) for solid treatment-related SMN. High cummulative doses of ifosfamide, cyclophosphamide, and etoposide were most frequently encountered among study patients.

**Conclusions:**

Patients with neuroblastoma are at more risk of developing hematological than solid treatment-related SMN after relatively longer duration for latter compared to former tumor subtypes. High-risk treatment regimens and higher cumulative doses of alkylating agents and Topoisomerase-II inhibitors are likely associated with increased risk of treatment-related SMN.

## Introduction

Neuroblastoma (NB) is a rare disease representing about 8–10% of childhood cancers and is the most common extra-cranial solid tumor in children [1, 2]. Treatment related secondary malignant neoplasm (tr-SMN) development is a known side effect with cumulative incidence at 10 and 30 y of 1.8% and 10.44%, respectively in high-risk (HR) NB versus much lower incidence in low (LR) and intermediate-risk (IR) patients at 30 y [3.57%; (P < 0.001)] [3–5].Over the past two decades, long term survival of patients with NB was thoroughly investigated by the Children’s oncology Group (COG), trying to reduce treatment in order to minimize treatment-related toxicities and side effects like SMNs as much as possible. Both LR and IR treatment intensity was successfully reduced while keeping the same excellent outcomes; the 5-y event free survival (EFS) and overall survival (OS) were respectively 90.7% and 97.9% in LR and 85.1% and 95.8%, in IR-NB [6]. On the contrary, patients with HR-NB had poorer prognosis on standard therapy which was significantly improved from less than 20% survival rates in the 1980s [6]. Despite the steadily improved disease outcome as reported by COG; intensifying treatment by using multi-modality treatment approaches combining intensive induction chemotherapy, high dose myeloablative consolidation with autologous (single/tandem) bone marrow transplantation (ABMT) followed by radiotherapy, differentiating agents, and immunotherapies, resulted in only 5-y EFS and OS rates of 57.2% and 62.5%, respectively [6, 7].

Different tr-SMNs were reported to arise at different latency periods and incidence rates post treatment of NB with different risk groups, associated with certain treatment agents or occasionally with family cancer history, showing hematological and solid tr-SMNs rates of 0.35% and 0.85%, respectively [3]. The most frequently encountered NB therapy-related SMNs were acute myeloid leukemia (AML), myelodysplastic syndrome (MDS), acute lymphoblastic leukemia (ALL) and chronic myeloid leukemia (CML) [8]. Solid SMNs were also associated with radiotherapy, depending on the age of patient during treatment, dose and site of radiation [8]. Cumulative dose, intensity and duration of contributing chemotherapeutic agents were also reported as important risk factors [9].

Combined intensive chemotherapeutic regimens in NB treatment including alkylating agents (e.g. cyclophosphamide, ifosfamide, melphalan, busulfan), topoisomerase-II inhibitors (e.g. etoposide, doxorubicin) and platinum-based drugs (e.g. cisplatin and carboplatin), in addition to radiation therapy, increased the risk of tr-SMNs [6, 7].

Topoisomerase-II inhibition leads to DNA breakage at fragile spots, with frequent improper repairs of these breaks, chromosomal translocations such as t(11q23;var) on the MLL gene and likely on other leukemia-associated genes (e.g. RUNX1, etc.) allowing these hematopoietic stem cells (HSCs) to escape apoptosis and lead to clonal expansion and the development of therapy-related leukemias. Similarly, alkylating DNA bases leads to mutations in TP53 (tumor suppressor gene), RUNX1, P53, and RAS pathways which regulate cell cycle and apoptosis. Monosomy 5, monosomy 7, and 5q- or 7q- deletions are also associated with alkylating agents along with long-term genomic instability causing therapy-related myeloid neoplasms, leukemias and solid tumors [10].

The aim of the present study was to evaluate the long-term effect of multi-modal, risk-based treatment protocols on the development of tr-SMNs in patients during or after treatment of NB at Children’s Cancer Hospital-Egypt (CCHE).

## Material and methods

This retrospective, descriptive study included all patients with NB treated at CCHE during the period from July 2007 to December 2022. Patients were enrolled on either COG A3973 or Neuroblastoma Study Group of the Société Française d'Oncologie Pédiatrique (SFOP) protocols. Informed consents from patients’ legal guardians according to Helsinki declaration [11] and institutional review board (IRB) approval were obtained prior to study enrolment. All data was retrieved from the CCHE electronic medical records, using the REDCap (Research Electronic Data Capture) software.

Patients were pathologically classified according to the World Health Organization (WHO), International Classification of Disease for Oncology (ICD-O)-3rd edition, as confirmed non-central malignant NB (ICD-O-3 = 9500) or Ganglioneuroblastoma (GNB) (ICD-O-3 = 9490) [12]. Initial disease staging followed the International Neuroblastoma Staging System (INSS) criteria and risk stratified by the COG risk classification system [7, 13]. Eligible patients must had confirmed SMNs of any type either during or after treatment with risk-adapted NB protocols.

Collected data included patient’s history, clinical manifestations and patient’s demographics (gender and age at NB diagnosis). Initial radiological images of the primary tumor site and metastatic sites were reviewed for proper staging using positron emission tomography (PET) scan/meta-iodobenzylguanidine (MIBG) scan, computed tomography (CT) with contrast and magnetic resonance imaging (MRI). Other workup evaluation included bilateral bone marrow aspirate (BMA) and biopsy (BMB), histopathology classification with degree of differentiation of tumor cells using the International Neuroblastoma Pathology Criteria (INPC) guidelines [14]. The MYCN gene amplification was tested using Fluorescence In Situ Hybridization (FISH) [15]. Prior to the development of tr-SMNs, cumulative doses of different chemotherapeutic agents administered per regimen during different protocol phases and salvage treatment were calculated, (Table 1), then total cumulative doses of chemotherapeutic agents and their average were calculated based on number of regimen cycles given per each patient.

**Table 1:** Chemotherapeutic agents, regimens and cumulative doses per regimen used in neuroblastoma treatment protocols

Treatment phase	Regimen	Chemotherapeutic agents with cumulative doses per regimen
Induction	CDV	Cyclophosphamide (4200 mg/m^2^)/doxorubicin (75 mg/m^2^)/vincristine (2mg/m^2^)
	CiE	Cisplatin (200 mg/m^2^)/etoposide (600 mg/m^2^)
	CADO	Cyclophosphamide (1500 mg/m^2^)/doxorubicin (60 mg/m^2^)/vincristine (3 mg/m^2^)
	VP16/Carbo	Etoposide (600 mg/m^2^)/carboplatin (450 mg/m^2^)
Maintenance	Topo/Cyclo	Topotecan (3.75 mg/m^2^)/cyclophosphamide (1250 mg/m^2^)
	OJEC	Vincristine (1.5 mg/m^2^)/carboplatin (500 mg/m^2^)/etoposide (200 mg/m^2^)/cyclophosphamide (600 mg/m^2^)
	OPEC	Vincristine (1.5 mg/m^2^)/cisplatin (100 mg/m^2^)/etoposide (200 mg/m^2^)/cyclophosphamide (1200 mg/m^2^)
Second line (salvage)	ICE	Ifosfamide (9000 mg/m^2^)/carboplatin (450 mg/m^2^)/etoposide (500 mg/m^2^)
	TEMIRI	Temozolamide (500 mg/m^2^) /irinotecan (100 mg/m^2^)
Consolidation pre SCT^a^	Bu/Mel	Busulfan (20mg/kg)/melphalan (140 mg/m^2^)

^a^*SCT* stem cell transplant

### Statistical methods

Numerical variables were presented as mean, median and range while categorical variables were expressed as frequencies and percentages. Statistical analysis was applied using IBM SPSS (Statistical Package for the Social Science) Statistics, version 20. Specifically, the Kaplan–Meier method was used to estimate patient’s OS, comparison was done using the log-rank test. The OS was calculated from time of enrollment on NB protocols till time of death due to any cause or till the date of last contact in alive patients. Survival estimates were reported as 5-y survival ± SE (Standard Error) with statistical significance set as p < 0.05. Fisher’s exact test was used to compare NB diagnostic characteristics and survival status of patients with secondary malignancies. The latency period to development of tr-SMNs was calculated from date of NB diagnosis till tr-SMN event and the median survival time was estimated since date of development of tr-SMNs till date of death or last follow up.

## Results

**Fig. 1:**
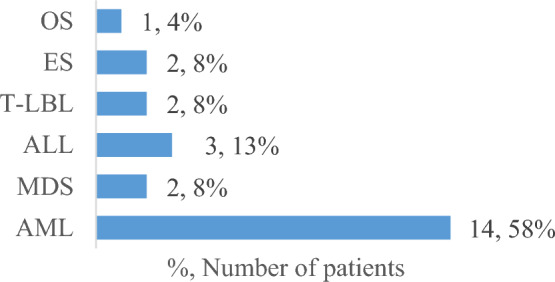
Study patients’ distribution by tr-SMN disease subtype. *OS* osteosarcoma, *ES* ewing sarcoma, *T-LBL* T-cell lymphoblastic lymphoma, *ALL* acute lymphoblastic leukemia, *MDS* myelodysplastic syndrome, *AML* acute myeloid leukemia

A total of 2514 patients were presented with NB during the period from July 2007 to December 2022. All patients reviewed were 2,290 out of the total number [excluding patients with ganglioneuroma, patients with no intervention on watch and see protocol and surgery only treated patients] received risk-adapted, multimodal treatment protocols and followed up within a range from 2 to 16 y. Twenty-four; 13 males and 11 females (male/female, 1.2:1) suffered from either hematological or solid tr-SMNs (Fig. 1) during or after treatment of their primary NB disease, representing 1% of all enrolled patients during the study period. Age of the 24 study patients ranged from 6 mo to 9.5 y at time of diagnosis of NB (29% were < 1 y, 13% 1–1.5 y and the majority (58%) were ≥ 1.5 y; median: 2 y). Except for only one patient with thoracic NB, 23/24 presented initially with abdominal tumor (13 adrenal and 10 non-adrenal). Initially metastatic stage 4 NB found in 58% (80% with BM infiltration) versus stage 3 in 42% of the study patients, (Table 2).

**Table 2:** Initial characteristics of neuroblastoma disease treated study patients

Variables		Patients with secondary malignant neoplasm			P-value^b^
		Total (%) (n = 24)	Alive	Dead	
Demographic and clinical criteria					
Age	< 1 y	7 (29)	4	3	0.516
	1 to < 1.5 y	3 (13)	2	1	
	≥ 1.5 y	14 (58)	5	9	
Gender	Female	11 (46)	5	6	1.00
	Male	13 (54)	6	7	
Primary site	Thoracic	1 (4)	0	1	1.00
	Abdominal	23 (96)	11	12	
	Adrenal	13	5	8	0.632
	Retroperitoneal	4	2	2	
	Para-spinal	6	4	2	
Stage	Stage 3	10 (42)	5	5	1.00
	Stage 4	14 (58)	6	8	
Risk stratification	High risk	16 (67)	5	11	0.082
	Intermediate risk	8 (33)	6	2	
Pathological features					
Degree of differentiation	Differentiating	1 (4)	1	0	0.724
	Undifferentiated/poorly differentiated	20 (83)	9	11	
	Not applicable	3 (13)	1	2	
INPC^a^	Favorable Shimada	3 (13)	2	1	0.590
	Unfavorable Shimada	20 (83)	9	11	
	Not applicable	1 (4)	0	1	
Histological subtype	Ganglioneuroblastoma	2 (8)	1	1	1.00
	Neuroblastoma	22 (92)	10	12	
MYCN amplification	Amplified	4 (17)	1	3	0.596
	Not amplified	20 (83)	10	10	

^a^INPC, International Neuroblastoma Pathology Criteria

^b^The effectiveness of the patients’ initial characteristics against their survival status; significance set as p < 0.05

**Fig. 2:**
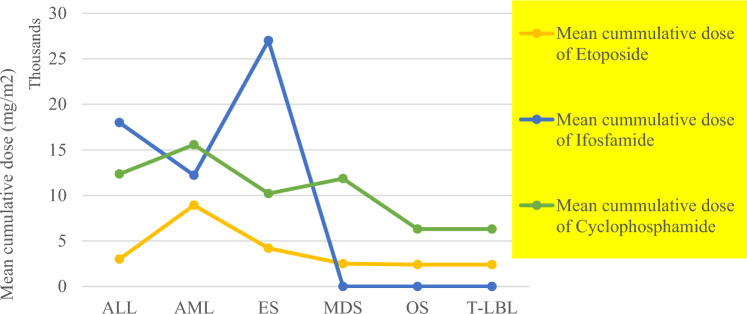
Mean cumulative doses (mg/m^2^) of etoposide, cyclophosphamide and ifosfamide by tr-SMN disease subtype, *OS* osteosarcoma, *ES* ewing sarcoma; *T-LBL*: T-cell lymphoblastic lymphoma, *ALL* acute lymphoblastic leukemia, *MDS* myelodysplastic syndrome, *AML* acute myeloid leukemia

Among all study patients, 16/24 (67%) were treated on HR protocol (11 received CDV/CIE and 5 VP16/Carbo alternating with CADO induction therapy), while 8/24 (33%) were treated as IR with VP16/Carbo alternating with CADO regimen (7/8 patients received 8 cycles). Respectively; 10/16 and 1/8 of HR and IR-treated patients, were given 2nd and/or 3rd lines of therapy due to recurrent, progressive or stationary NB. Autologous stem cell transplantation done for almost 50% of HR patients. All patients with solid tr-SMNs didn’t receive radiotherapy on site of SMN before. On the contrary, 52% of patients with hematological tr-SMNs received radiotherapy (9 at local sites, 1 at metastatic site and 1 at both local and distant site), at a range of 2160–3600 cGy. Calculated average total cumulative chemotherapy doses received prior to the occurrence of SMNs for etoposide, cyclophosphamide and ifosfamide were respectively, 6.4 g/m^2^ (1.2–34.8 g/m^2^), 13.2 g/m^2^ (4.5–24.3 g/m^2^) and 11.6 g/m^2^ (0.0–54 g/m^2^), (Fig. 2) based on the cumulative doses of chemotherapeutic agents per each regimen (Table 1) and the number of cycles received per each patient. The highest cummulative doses of ifosfamide and etoposide were associated with ES and AML as tr-SMNs, respectively. while highest cummulative dose of cyclophosphamide was encountered with AML, MDS and ALL, (Fig. 2).

**Fig. 3:**
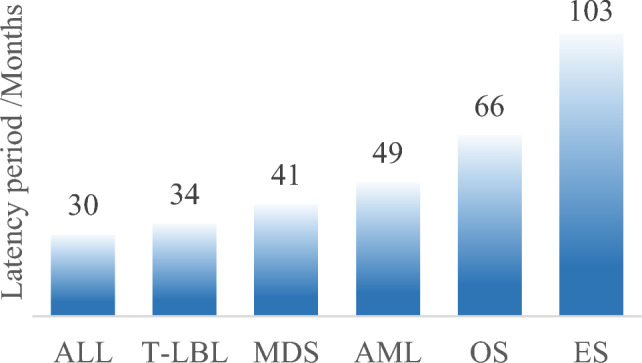
Mean latency time from NB diagnosis to tr-SMNs development/months. *OS* osteosarcoma, *ES* ewing sarcoma, *T-LBL* T-cell lymphoblastic lymphoma, *ALL* acute lymphoblastic leukemia, *MDS* myelodysplastic syndrome, *AML* acute myeloid leukemia

Estimated time period to development of tr-SMNs showed a variable wide range with shorter mean and median durations noticed for hematological compared to solid tr-SMNs yet it was not statistically confirmed; 14 mo to 8.3 y (mean: 3.7 y, median: 3.3 y) versus 5.5 to 9.2 y (mean: 7.6 y, median: 8 y) respectively (Fig. 3).

By end of the study, 11/24 (46%) of our patients remained alive (9 hematological and 2 solid tr-SMNs) with 5-year OS of 60.3% ± 18.3%. On the contrary, 13 patients (54%) died; 7/13 within average of 7 mo (ranging from 3 to 13 mo) since their tr-SMNs development; 5/13 at the early beginning of SMNs treatment and 1/13 due to progressive NB disease post treatment of AML. The estimated median survival time for all patients was 13.1 months since development of tr-SMNs.

## Discussion

The long experience of CCHE with NB treatment between July 2007 and December 2022 has been reviewed in this study to explore the prevalence of tr-SMNs development among patients primarily treated with different risk-based NB protocols. The incidence of tr-SMNs among the whole study population of NB found to be 1% which is very close to other studies within the enrollment years between 1973 and 2015; ranging from 0.77% to 1.2% [3, 4, 16]. Our data also showed close distribution by sex with only slightly higher incidence in males than females (1.2:1) which was also comparable to other cohorts [3, 16], and unlike some others that sometimes showed relatively less common tr-SMNs in males than females (1:1.7) [4] and (1:3) [17].

Risk factors commonly observed with tr-SMNs in our study were also found similar to other reports from France, United Kingdom and the United States of America; including age of NB diagnosis ≥ 18 mo [4, 16, 17], primary abdominal site [16, 17], and INSS stage 4 disease [3, 4]. Histopathological subtype with unfavorable, undifferentiated or poorly differentiated, NB was evidenced in almost all study cohort that was consistent to previously published results by Applebaum et al. [4]. The MYCN gene amplification, known surrogate of aggressive NB and bad prognosis [18], was not encountered in most of our study cohort (83%). Same finding was found in previous other reports [4]. However, none of the above-mentioned factors showed any statistically significant difference on survival status between the corresponding subgroups. This could be related to the small number of participants in this cohort.

Along the years and through different eras of NB management with dynamically changing concepts, the occurrence of tr-SMNs was usually a risk [16]; in the US SEER database, 23 out of 38 patients with NB treated between 1973 and 1989 developed tr-SMNs while only 5/38 patients were between 1990 till 1999 [16]. This was more or less correlated with the various alterations in NB treatment since the 1970s, including adoption of risk-based stratification, where the intensity of therapy was reduced for low- to intermediate-risk patients and more intensified for high-risk patients. Our study patients (n = 24) were subjected to almost same treatment strategies during the study period between 2007 and 2022. Of our patients, 16/24 (67%) developed tr-SMNs on top of treatment of their HR-NB and close to incidence of 62.5% reported by other investigators. On the contrary, 8/24 (33%) of our tr-SMNs patients were treated on low/intermediate risk NB protocols, in consistence to reported 37.5% in other cohorts with similar risk groups [4].

In our study, 18/24 tr-SMNs patients were diagnosed as NB between 2007 and 2015, whereas only 6/24 tr-SMNs patients were initially diagnosed since 2016 till present, this difference could be explained by the cumulative increase over the extended follow up time elapsed for the earlier diagnosed patients. Hematological tr-SMNs (n = 21), specially AML found to occur with an average of 6 y from initial diagnosis of NB, while for solid tr-SMNs, ES took up to around 10 y to develop_._ Similarly, most of studies reported the generally increased risk for tr-SMNs occurrence during the first 10 y from initial diagnosis of NB and longer time to solid than hematological tumor development [3, 4, 16, 19]. Extended follow-up is thus required to diagnose more solid tumors among survivors, yet, considering the lower survival rates among most of patients with HR-NB patients, this could not be feasible to track.

In search of possible risk factors, we observed the cumulative dose of some chemotherapeutic agents with tr-SMNs development where higher cumulative doses of etoposide, ifosfamide and cyclophosphamide were reported in most, but not all study patients, (Fig. 2). Radiotherapy administered in about 50% of our patients while their SMNs occurred at different sites other than radiation exposure sites. Although one of our patients developed tr-SMNs early during the course of NB therapy, most patients developed late tr-SMNs were found among relapsed and progressive NB which is probably linked to prolonged chemotherapy. Moreover, patients with LR-NB who were not exempted of the risk of developing SMNs as shown by other investigators [3, 4, 16].

Variability of risk factors and diversity of SMNs development, could be ought to the presence of other underlying tr-SMNs predisposing factors. In support of this opinion is the presence of other patients who developed the same tr-SMNs without being exposed to similar therapy conditions. The potential involvement of underlying genetic as well as other biological factors warrant further studying of tr-SMNs susceptibility.

## Conclusions

Patients with NB are at more risk of developing treatment related hematological, specially AML, than solid tr-SMNs after relatively longer duration for latter compared to former tumor subtypes. High-risk treatment regimens and higher cumulative doses of alkylating agents and Topoisomerase-II inhibitors are likely associated with increased risk of tr-SMNs. Studying the genetic and biological background of susceptible patients might reveal other underlying risk factors.

## Data Availability

The datasets and all relevant raw data generated during and/or analyzed during the current study are available from the corresponding author upon request.
